# Neurocysticercosis: An Uncommon Cause of New-Onset Seizure in the United States

**DOI:** 10.7759/cureus.65864

**Published:** 2024-07-31

**Authors:** Surender Singh, Taylor E Browning, Naina S Jakhar

**Affiliations:** 1 Pulmonary and Critical Care, Ascension Health, Indianapolis, USA; 2 Internal Medicine, Ascension Health, Indianapolis, USA; 3 Biology, Carmel High School, Carmel, USA

**Keywords:** ct head, dexamethasone, albendazole, seizure, neurocysticercosis

## Abstract

Neurocysticercosis is predominantly a disease in tropical countries. However, with increasing migration, it is now more frequently reported in developed countries as well. We are reporting a case of new-onset seizures due to underlying neurocysticercosis in a 31-year-old male migrant patient. Initial imaging revealed two cystic lesions in the left parietal lobe and another small lesion in the right parietal lobe. The imaging findings were highly suggestive of neurocysticercosis. Our case highlights the important issue of neurocysticercosis as an etiology for new-onset seizures in the United States.

## Introduction

Neurocysticercosis is a parasitic infection impacting above 50 million people around the world and is more prevalent in certain regions such as South Asia, sub-Saharan Africa, and Central and South America [1,2]. It is one of the most frequent preventable causes of epilepsy worldwide, accounting for approximately 30% of new epilepsy cases in endemic regions [3]. However, it is an uncommon infection in the United States. Due to continuous migration across the world, neurocysticercosis has become increasingly prevalent and more often detected throughout the United States. Over 2,300 patients are hospitalized due to neurocysticercosis yearly, and approximately 2% of the new onset of seizure cases are linked to this infection [4,5]. To emphasize the significance of considering neurocysticercosis as a differential diagnosis in cases of new-onset seizures, particularly in regions with significant immigrant populations from endemic areas.

## Case presentation

**Figure 1:**
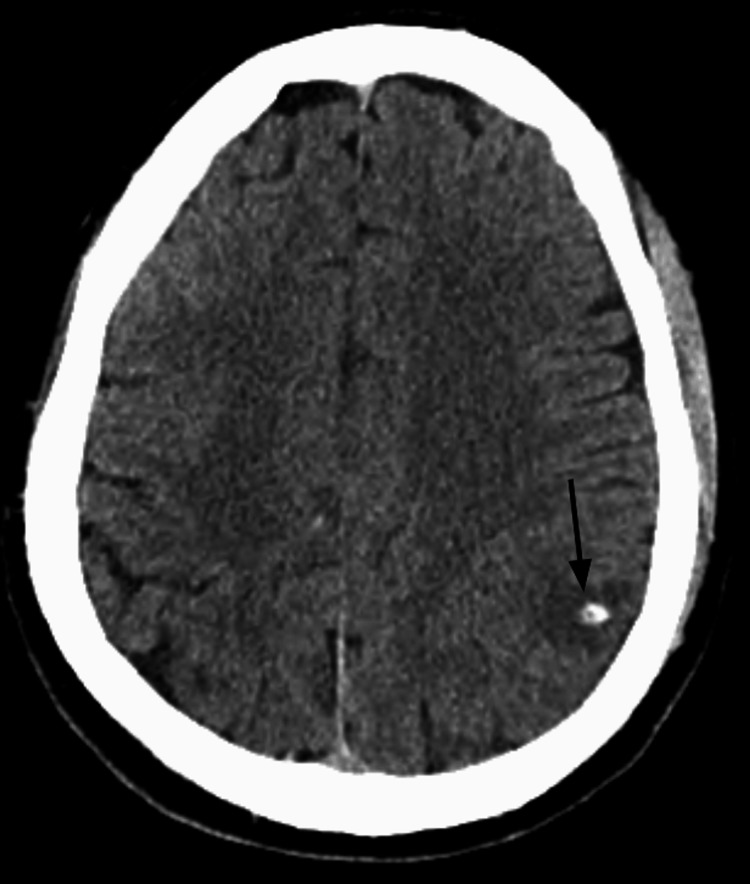
CT head reveals a cystic lesion in the mesial left parietal lobe with peripheral calcification, indicative of a scolex (vesicular stage)

**Figure 2:**
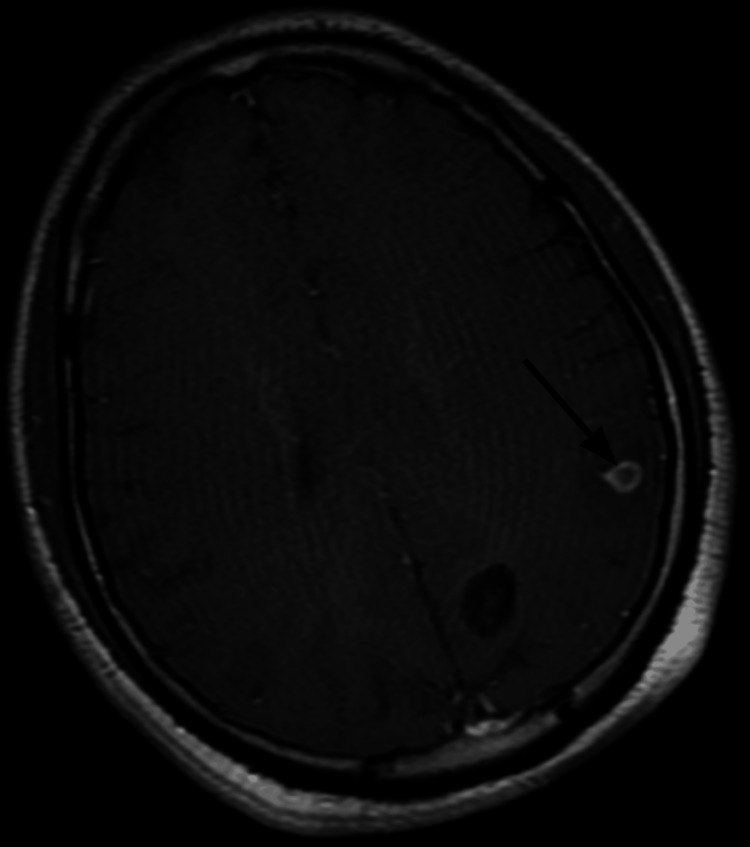
MRI of the brain T1 sequence confirms the presence of a cystic lesion in the left parietal lobe

**Figure 3:**
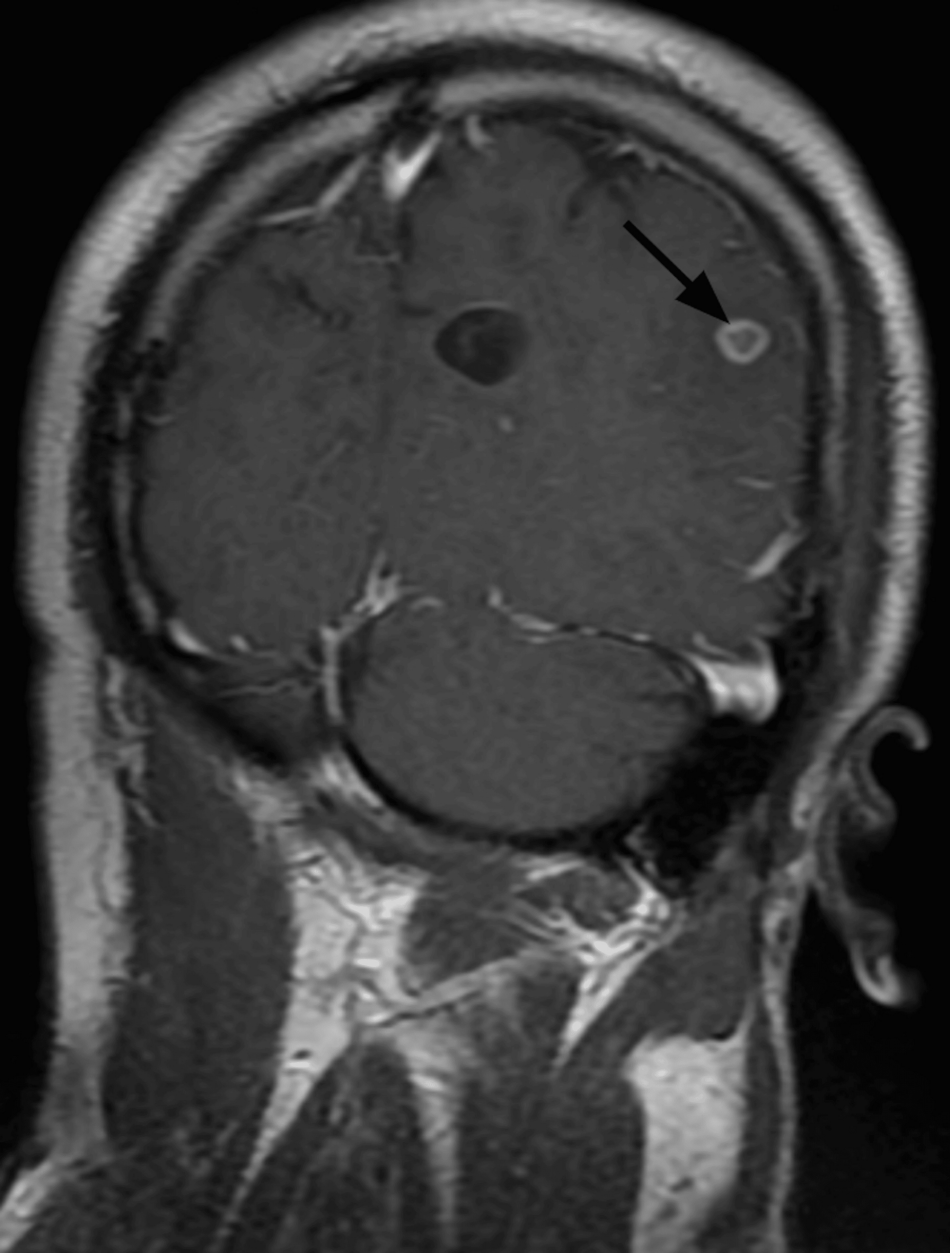
MRI of the brain in the coronal plane confirms the presence of a cystic lesion in the left parietal lobe

**Figure 4:**
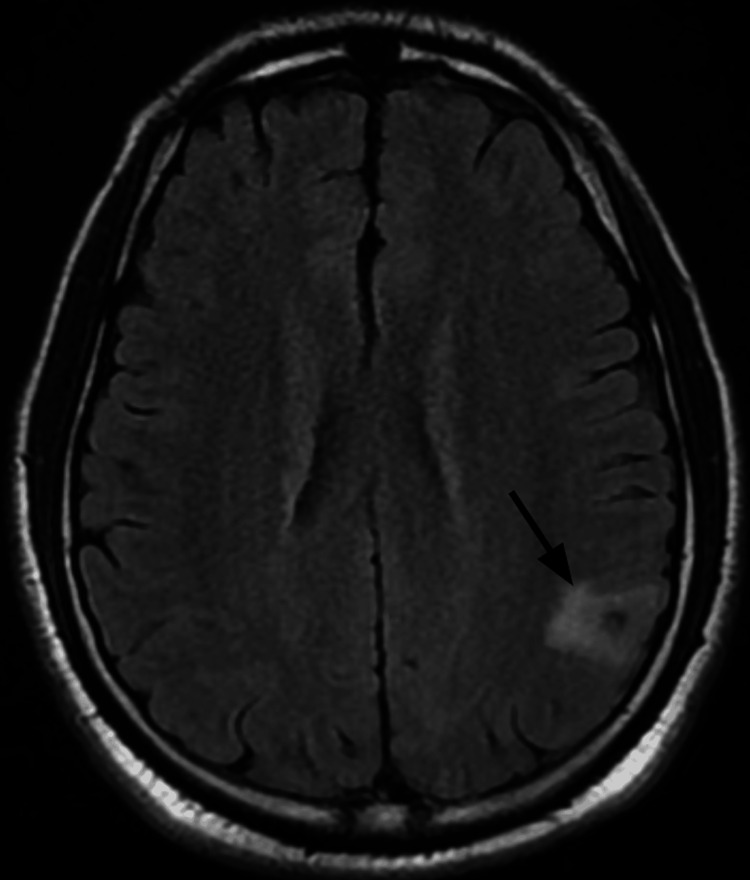
T2 FLAIR MRI of the brain reveals a second lesion in the left parietal lobe surrounded by vasogenic edema FLAIR: Fluid-attenuated inversion recovery

A Haitian male, 31 years of age, without any significant past medical history arrived at the hospital with new-onset seizures. The patient had immigrated from Haiti about two years ago and was in his usual state of health before experiencing a three-minute seizure at home. The patient was actively seizing upon arrival at the emergency department, which was aborted with Lorazepam. He was subsequently loaded with Levetiracetam. Once stabilized, the exam of the patient was unremarkable except for his postictal state. Patient laboratory test results were normal except for mild lactic acidosis, which was likely secondary to multiple seizures, resolved with appropriate fluid resuscitation and cessation of the seizures (Table 1). Of note, urine drug screen and HIV testing were negative. Stool testing for ova and parasites was negative. A CT head revealed three lesions suggestive of neurocysticercosis, and an MRI was ordered for better characterization. The first active cystic lesion, located within the mesial left parietal lobe, demonstrated calcifications most consistent with a scolex or vesicular stage lesion (Figures 1-3). The second lesion within the left parietal lobe displayed surrounding vasogenic edema, representing an active lesion (Figure 4). Finally, there was a punctate calcification in the mesial right parietal lobe consistent with a nodular stage lesion. With neurocysticercosis suspected, infectious disease, neurology, and neurosurgery services were consulted. Diagnosis of cysticercosis was made based on classic brain Imaging findings and epidemiological exposure from endemic areas in the past. CSF analysis was not required. The patient was started on dexamethasone and albendazole 600 mg twice daily for 10 days, initiated once steroids had been on board for at least 24 hours. Due to the location of the cysts, neurosurgical intervention was not indicated. The patient continued to recover well in the hospital and denied any symptoms except for a mild headache. An EEG did not reveal any additional seizure activity. He was discharged home on day 4 of hospitalization on albendazole, dexamethasone, and levetiracetam with close outpatient follow-up.

**Table 1:** Laboratory test results

Test name	Result	Normal range	Units
Hemoglobin	15.3	12.8-16.9	g/L
White blood cell count	8.3	3.3-10.5	K/CUMM
Platelet count	310	150-450	K/CUMM
Sodium	140	135-145	mmol/L
Potassium	4.1	3.5-5.0	mmol/L
Chloride	106	98-110	mmol/L
CO2	26	22-30	mmol/L
Blood urea nitrogen	19	9-20	mg/dL
Creatinine	1	0.66-1.20	mg/dL
Glucose	112	60-99	mg/dL
Calcium	9.2	8.4-10.2	mg/dL
Lactic acid	1.21	0.36-0.75	mmol/L

## Discussion

Neurocysticercosis is caused by the parasite *Taenia solium* and is usually spread via ingestion of *T. solium* eggs [6]. It is endemic in a large part of the developing world, including Haiti. Within these regions, *T. solium* is likely the most frequent cause of acquired seizures and has significant neurologic morbidity. Recognizing cysticercosis can be difficult in countries outside of endemic parts of the world due to the lack of specific findings on the routine blood workup. Diagnosis is typically based on clinical signs and symptoms, imaging results, and prior exposure history. The most common symptoms are seizures and headaches. Patients can also report changes in vision, confusion, focal neurological signs, and rarely stroke and meningitis, but fever is not a typical hallmark. Seizures are considered to be the most frequent symptom in parenchymal neurocysticercosis, while headaches and intracranial hypertension are more common in extra-parenchymal disease [7]. Diagnosis is made through a combination of serologic and radiographic findings. In those with active brain parenchymal disease on imaging, the mainstays of therapy include corticosteroids and albendazole for 10-14 days. Praziquantel is added if there are more than two active lesions [8-10]. Antiepileptics are also recommended for seizure prevention for at least six months or until the resolution of brain lesions on follow-up imaging. Follow-up brain imaging is usually suggested after six months of initial antiparasitic treatment, and then every six months until the complete resolution of cystic lesions. Even though neurocysticercosis is not endemic in the United States, the large immigrant population necessitates, considering it in the differential diagnosis for new-onset seizures.

## Conclusions

Neurocysticercosis, caused by the tapeworm *T. solium*, is a leading cause of acquired seizures in endemic regions of the world. While rare in the United States, increasing migration from endemic areas has led to a rise in reported cases. Our case underscores the significance of considering neurocysticercosis as a differential diagnosis in cases of new-onset seizures, particularly among immigrants or travelers to endemic regions, to ensure timely and accurate diagnosis and treatment.
